# Clinical outcomes of stage I and IIA non-small cell lung cancer patients treated with stereotactic body radiotherapy using a real-time tumor-tracking radiotherapy system

**DOI:** 10.1186/s13014-016-0742-3

**Published:** 2017-01-05

**Authors:** Norio Katoh, Itaru Soda, Hiroyasu Tamamura, Shotaro Takahashi, Yusuke Uchinami, Hiromichi Ishiyama, Kiyotaka Ota, Tetsuya Inoue, Rikiya Onimaru, Keiko Shibuya, Kazushige Hayakawa, Hiroki Shirato

**Affiliations:** 1 Department of Radiation Oncology, Hokkaido University Hospital, North-14 West-5, Kita-ku, Sapporo, Japan; 2 Global Station for Quantum Medical Science and Engineering, Global Institution for Collaborative Research and Education (GI-CoRE), Hokkaido University, Sapporo, Japan; 3 Department of Radiology and Radiation Oncology, Kitasato University School of Medicine, Sagamihara, Japan; 4 Department of Nuclear Medicine, Fukui Prefectural Hospital, Fukui, Japan; 5 Department of Therapeutic Radiology, Yamaguchi University Graduate School of Medicine, Ube, Japan; 6 Department of Radiation Medicine, Hokkaido University Graduate School of Medicine, Sapporo, Japan

**Keywords:** Stereotactic body radiotherapy, Non-small cell lung cancer, Real-time tumor-tracking radiotherapy, Gated radiotherapy, Image-guided radiotherapy

## Abstract

**Purpose:**

To investigate the clinical outcomes of stage I and IIA non-small cell lung cancer (NSCLC) patients treated with stereotactic body radiotherapy (SBRT) using a real-time tumor-tracking radiotherapy (RTRT) system.

**Materials and methods:**

Patterns-of-care in SBRT using RTRT for histologically proven, peripherally located, stage I and IIA NSCLC was retrospectively investigated in four institutions by an identical clinical report format. Patterns-of-outcomes was also investigated in the same manner.

**Results:**

From September 2000 to April 2012, 283 patients with 286 tumors were identified. The median age was 78 years (52–90) and the maximum tumor diameters were 9 to 65 mm with a median of 24 mm. The calculated biologically effective dose (10) at the isocenter using the linear-quadratic model was from 66 Gy to 126 Gy with a median of 106 Gy. With a median follow-up period of 28 months (range 0–127), the overall survival rate for the entire group, for stage IA, and for stage IB + IIA was 75%, 79%, and 65% at 2 years, and 64%, 70%, and 50% at 3 years, respectively. In the multivariate analysis, the favorable predictive factor was female for overall survival. There were no differences between the clinical outcomes at the four institutions. Grade 2, 3, 4, and 5 radiation pneumonitis was experienced by 29 (10.2%), 9 (3.2%), 0, and 0 patients. The subgroup analyses revealed that compared to margins from gross tumor volume (GTV) to planning target volume (PTV) ≥ 10 mm, margins < 10 mm did not worsen the overall survival and local control rates, while reducing the risk of radiation pneumonitis.

**Conclusions:**

This multi-institutional retrospective study showed that the results were consistent with the recent patterns-of-care and patterns-of-outcome analysis of SBRT. A prospective study will be required to evaluate SBRT using a RTRT system with margins from GTV to PTV < 10mm.

**Electronic supplementary material:**

The online version of this article (doi:10.1186/s13014-016-0742-3) contains supplementary material, which is available to authorized users.

## Background

Surgical resection is the standard of care for patients with early stage non-small cell lung cancers (NSCLC). However, patients with early stage NSCLC often cannot tolerate surgical resection because of age and/or co-morbidities such as chronic obstructive pulmonary and cardiac diseases. With the advent of stereotactic body radiation therapy (SBRT) and appropriate image-guidance in the radiotherapy, it is now possible to administer very high radiation doses to peripheral early stage NSCLC over a short treatment time period without high risks of complications [1–5]. Recent multivariable analysis has shown improved overall survival with SBRT compared with patients who received no treatment (hazard ratio, 0.64; p < .001) [6]. The SBRT is now recommended for patients with early stage NSCLC who are medically inoperable or refuse surgery [7]. Whether SBRT should be the first choice of treatment for high risk patients rather than surgical resection is still to be determined in a prospective randomized trial [8–10].

Since inoperable early stage NSCLC patients often suffer from poor respiratory functions, it is critically important to reduce the irradiated volume to normal lung tissue in the treatment of lung tumors with SBRT, and there have been many investigations to reduce the uncertainty of tumor location due to respiration [11–14]. Inadequate respiratory motion management in SBRT has been one of the causes of local recurrences [15]. Motion management is essential in SBRT of lung tumors to be able to deliver the treatment dose accurately. Four-dimensional treatment planning, gating with a linear accelerator, and real-time tracking of the internal tumor motion have been shown to reduce the uncertainties due to respiratory motion [16–18]. In 1999, a real-time tumor-tracking radiotherapy (RTRT) system was developed and put into use for SBRT. This RTRT system uses two sets of fluoroscopes in the treatment room for the real-time tracking of the internal fiducial markers implanted in or near the lung tumor, tracking at 30 times a second [18]. In the system, the linear accelerator is gated to irradiate the tumor only when the implanted fiducial marker is within 2 mm of the planned position which has been determined in 4days treatment planning [19]. Although the RTRT system was introduced in several institutions in Japan and has been used for the SBRT of early stage NSCLC for a decade, clinical results were reported from only one institution [20–22] and no multi-institutional clinical results of SBRT using the RTRT system have been published.

The purpose of the present study is to evaluate clinical results of SBRT for stage I and IIA NSCLC used with this RTRT system in four institutions in Japan over the last twelve years by patterns-of-care and patterns-of-outcome analysis.

## Methods

### Patients

We obtained written informed consent to administer SBRT using the RTRT system from all patients and approval from the institutional review boards of all four institutions for this retrospective study. We reviewed patients treated with SBRT using the RTRT system from September 2000 to April 2012, diagnosed with histologically proven NSCLC and peripherally located clinical stage I and IIA as determined by the seventh edition of the Union for International Cancer Control staging criteria. A peripherally located tumor was defined as a tumor located outside a volume 2 cm in all directions around the proximal bronchial tree. In principle, all the patients with histologically proven, peripherally located, stage I and IIA NSCLC were treated by SBRT using RTRT in the four institutions during this period. Patients were excluded from the study if they had received thoracic radiation therapy for simultaneous malignant tumors within three months before or after the start dates of the SBRT.

A total of 283 patients with 286 tumors were identified. The median age was 78 years (52–90). Among the 286 tumors, 155, 67, 41, and 23 tumors were treated in the four institutions. The maximum tumor diameters were from 9 to 65 mm with a median of 24 mm. Patient characteristics are detailed in Table 1.

**Table 1:** Patient characteristics and treatments

	n	Adenocarcinoma	SCC
Patients	283		
Age (years)			
Median	78 (range 52–90)	79 (52–89)	77.5 (52–90)
Gender			
Male	214	124	74
Female	69	61	6
Performance status			
0	58	40	13
1	184	124	51
2	37	19	14
3	3	1	2
Unknown	1	1	0
Observation period (months)			
Median	28 (range 0–127)	30 (0–127)	25 (2–101)
Institutions			
A	155	97	45
B	67	47	16
C	41	25	14
D	23	16	5
Maximum tumor diameter (mm)			
Median	24 (range 9–65)	23 (9–65)	26 (13–65)
Number of tumors			
1	280	-	-
2	3	-	-
T stage			
1a and 1b	195	131	51
2a and 2b	91	54	29
Tumor location			
Right Upper Lobe	74	54	15
Right Middle Lobe	18	10	7
Right Lower Lobe	69	45	21
Left Upper Lobe	75	47	24
Left Lower Lobe	50	29	13
Operability			
Operable	71	49	16
Inoperable	190	120	55
Unknown	25	16	9

*SCC* squamous cell carcinoma

### SBRT using an RTRT system

The RTRT system has been described in detail elsewhere [19, 23]. In brief, the process for synchronizing the tracking of a marker with the irradiation was as follows. Before treatment, 1.5-mm or 2-mm gold markers were implanted near the tumor by bronchoscopy, principally within 5 cm of the center of the gross tumor volume (GTV). After the insertion of the fiducial markers, computed tomography was performed, usually while the patient held the breath at the end of expiration. The fluoroscopic RTRT system consists of four sets of diagnostic fluoroscopic, image-processor units, a trigger-control unit, an image-display unit, and a conventional linear accelerator with multileaf collimators. The linear accelerator is gated to irradiate the tumor only when the gold marker is within 2.0 mm of the planned coordinates relative to the isocenter in the lateral, craniocaudal, and anterior-posterior directions.

Patterns-of-care in SBRT using RTRT was retrospectively investigated in the four institutions. To evaluate the radiation dose, the biologically effective dose (BED) was calculated using the linear-quadratic model, defined as nd*(1 + d/(α/β)), where n is the number of fractions and d is the dose per fraction, assuming an α/β of 10 for tumors.

### Evaluation

Follow-up examinations and computed tomography (CT) scans were commonly performed every 3 to 6 months after the SBRT. The definition of local failure was as follows: sequential enlarging opacity for more than 6 months on CT images, enlarging opacity corresponding to FDG-PET abnormalities and/or histologic confirmation. Absence of local disease recurrence was defined as a locally controlled disease. Toxicities were assessed with Common Terminology Criteria for Adverse Events v3.0.

### Statistical analysis

The follow-up duration was calculated from the start date of the SBRT. The Kaplan-Meier method was used for calculating overall survival (OS) and local control (LC) rates. The log-rank test was used to compare subgroups. Multivariate analysis was performed using a Cox proportional hazards regression model. The hazard ratio (HR), 95% confidence interval (95%CI), and p value were calculated. The rates for Grade 2 or higher radiation pneumonitis were compared in subgroups using the univariate and multivariate logistic regression analyses where the odds ratio (OR) and 95%CI were estimated. A p value of < 0.05 was considered statistically significant. The JMP version 12 (SAS, Cary, NC) was used for the statistical analyses.

## Results

### Patterns-of-care in SBRT

Patterns-of-care in SBRT using RTRT are shown in Table 2. For the clinical target volume (CTV) margins, a margin of 0 mm was most frequently used. One institution usually adopted a CTV margin of 6 to 8 mm added to the GTV uniformly to include the microscopic tumor spread based on Giraud et al.’s report [24]. In another institution, a part of the GTV was expanded manually to ensure that the CTV included the tumor spiculations, which were not visualized on the planning CT images, but appeared on the diagnostic high-resolution CT images. Thus, all of 9 mm or wider CTV margins were delineated non-uniformly and adopted only in this institution, and were the maximum distance between the GTV and CTV contours. For the planning target volume (PTV) margin, which is comprised of the internal and the set-up margins, a margin of 5 mm was most frequently used. It varied from 3 to 12 mm depending on patient condition, the visibility of fiducial markers, and other factors. All SBRT plans were generated using three-dimensional conformal treatment planning techniques with a median of 6 static ports (range, 4–9). Thirty-nine treatment plans were calculated using the Clarkson method and 247 treatment plans used the Superposition method. A total dose of 35–60 Gy was administered in 4–9 fractions. The dose was prescribed at the isocenter in 189 treatment plans and the most frequent schedule was 48 Gy in 4 fractions in 149 treatment plans. The dose was prescribed for the 95% volume of the PTV (PTVD_95_) in 97 treatment plans and the most frequent schedule was 40 Gy in 4 fractions in 94 treatment plans. Among 286 treatment plans, a total of 234 treatment plans (137 prescribed at the isocenter and 97 prescribed to the PTV D_95_) were available for analysis of the dose to the PTVD_95_. The calculated BED (10) at the isocenter using the linear-quadratic model was from 66 Gy to 126 Gy with a median of 106 Gy in 286 treatment plans. The BED (10) to the PTVD_95_ was from 44 Gy to 106 Gy with a median of 80 Gy in 234 treatment plans.

**Table 2:** Patterns-of-care in SBRT using RTRT

	n	Adenocarcinoma	SCC
Number of ports			
4	2	1	1
5	89	64	21
6	173	101	56
7	17	14	2
8	4	4	0
9	1	1	0
X-ray energy (MV)			
4	4	4	0
6	231	148	64
10	51	33	16
CTV margin (mm)			
0	140	94	33
5	3	0	3
6	32	2	30
7	5	2	0
8	84	71	9
9	5	3	2
10	4	3	0
11	12	10	2
12	1	0	1
PTV margin (mm)			
3	67	47	16
5	112	70	34
7	17	11	4
8	74	47	22
10	11	6	3
12	1	1	0
unclassifiable	4	3	1
Margin from GTV to PTV (mm)			
0–4	67	47	16
5–9	57	37	13
10–14	110	66	38
15–	48	32	12
unclassifiable	4	3	1
Calculation algorithms			
Clarkson	39	29	10
Superposition	247	156	70
Prescription			
PTVD_95_	97	59	27
Isocenter	189	126	53
Dose (Gy/Fr)			
Prescription: PTVD_95_			
40/4	94	56	27
others	3	3	0
Prescription: Isocenter			
48/4	149	99	42
50/5	19	13	4
others	21	14	7
BED (10) at the Isocenter (Gy)			
≥ 100	205	134	55
< 100	81	51	25
BED (10) at the Isocenter (Gy)			
≥ 90	267	173	74
< 90	19	12	6

*BED* biologically effective dose, *CTV* clinical target volume, *Fr* fractions, *GTV* gross tumor volume, *Gy* gray, *MV* megavoltage, *PTV* planning target volume, *RTRT* real-time tumor-tracking radiotherpay, *SBRT* stereotactic body radiotherapy

### Overall survival and Local control

**Fig. 1:**
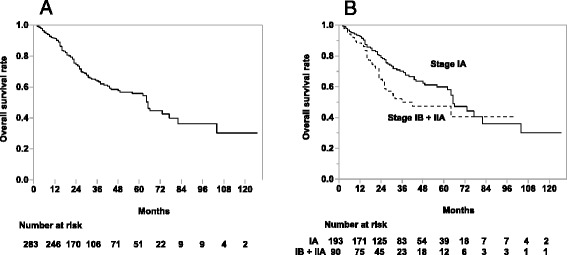
Overall survival rates in all patients (**a**), patients with stage IA and with stage IB + IIA (**b**), respectively

The median follow-up period was 28 months (range, 0–127). In the 283 patients, the OS rates for all patients, stage IA, and stage IB + IIA were 75%, 79%, and 65% at 2 years, and 64%, 70%, and 50% at 3 years, respectively (Fig. 1). There was no significant difference in OS rates among institutions. The results of the univariate analysis of OS rates are shown in Table 3. There was a statistically significant difference in the OS rates between the subgroups with females and males (3-year: 83% and 58%, respectively; *p* = 0.0016).

**Table 3:** Univariate and multivariate analysis results of overall survival rates

Variables		Overall Survival Rates			Univariate Analysis	Multivariate Analysis	
		n	2-year	3-year	*p*-value	HR (95%CI)	*p*-value
Stage	IA	193	79	70	0.0348	-	0.0693
	IB + IIA	90	65	50		1.484 (0.968–2.242)	
Histology	Adenocarcinoma	184	79	69	0.0689	-	0.5293
	SCC	79	69	52		1.151 (0.737–1.772)	
Gender	Female	69	86	83	0.0016	-	0.0015
	Male	214	71	58		2.393 (1.380–4.418)	
Location	Upper and Middle	165	76	65	0.925	-	0.9900
	Lower	118	73	62		0.997 (0.660–1.489)	
BED (10) IC (Gy)	≥ 90	264	75	65	0.4428	-	0.6830
	< 90	19	68	56		1.145 (0.570–2.083)	
Margins from GTV to PTV (mm)	≥ 10	156	72	63	0.3268	-	0.6307
	< 10	123	77	64		0.904 (0.596–1.355)	

*BED* biologically effective dose, *CI* confidence interval, *GTV* gross tumor volume, *HR* hazard ratio, *IC* isocenter, *OS* overall survival, *PTV* planning target volume, *SCC* squamous cell carcinoma

**Fig. 2:**
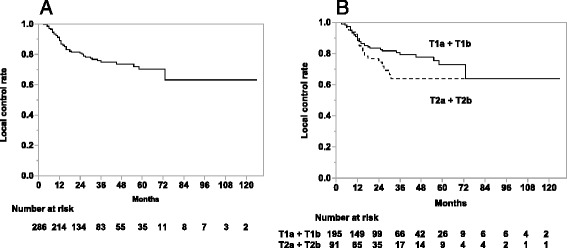
Local control rates of all tumors (**a**), T1a + T1b tumors and T2a + T2b tumors (**b**), respectively

In the 286 tumors, the LC rates for all tumors, T1a + T1b, and T2a + T2b tumors were 81%, 84%, and 74% at 2 years, and 75%, 79%, and 64% at 3 years (Fig. 2). There were no significant differences in the LC rates among institutions. The results of the univariate analysis of LC rates are shown in Table 4. There was a statistically significant difference in the LC rates between the subgroups with BED (10) at the isocenter ≥ 90Gy and BED (10) < 90 Gy (3-year: 78% and 42%, respectively; *p* = 0.0001), but no significant difference between BED (10) ≥ 100 Gy at the isocenter and BED (10) < 100 Gy (3-year: 74% and 76%, respectively; *p* = 0.8987, not listed in Table 4). There were no significant differences in the LC rates between BED (10) ≥ 80 Gy to the PTVD_95_ and BED (10) < 80 Gy (3-year: 81% and 72%, respectively; *p* = 0.1963, not listed in Table 4). Adenocarcinomas also showed more favorable LC rates than squamous cell carcinomas (SCC) (3-year: 80% and 62%, respectively; *p* = 0.0002). There were no significant differences in the OS and LC rates between margins from GTV to PTV ≥ 10 mm and margin < 10 mm, and also no significant differences in the OS and LC rates among the upper/middle and the lower lobes.

**Table 4:** Univariate and multivariate analysis results of local control rates

Variables		Local Control Rates (%)			Univariate Analysis	Multivariate Analysis	
		n	2-year	3-year	*p*-value	HR (95%CI)	*p*-value
T stage	T1a + T1b	195	84	79	0.1263	-	0.5903
	T2a + T2b	91	74	64		1.173 (0.647–2.076)	
Histology	Adenocarcinoma	185	87	80	0.0002	-	0.0044
	SCC	80	65	62		2.366 (1.311–4.301)	
Gender	Female	69	88	85	0.0595	-	0.2307
	Male	217	79	71		1.595 (0.751–3.700)	
Location	Upper and Middle	167	82	76	0.8487	-	0.8845
	Lower	119	79	73		1.042 (0.591–1.808)	
BED (10) IC (Gy)	≥ 90	267	84	78	0.0001	-	0.0017
	< 90	19	49	42		3.465 (1.655–6.706)	
Margins from GTV to PTV (mm)	≥ 10	158	81	71	0.8043	-	0.8723
	< 10	124	80	78		1.047 (0.589–1.833)	

*BED* biologically effective dose, *CI* confidence interval, *GTV* gross tumor volume, *HR* hazard ratio, *IC* isocenter, *LC* local control, *PTV* planning target volume, *SCC* squamous cell carcinoma

In the multivariate analysis, all clinical factors showed similar trends to those in the univariate analysis. In the OS rate, gender was a significant predictive factor (HR 2.393, 95%CI [1.380–4.418], *p* = 0.0015, Table 3). In the LC rate, significant factors were histology (HR 2.366, 95%CI [1.311–4.301], *p* = 0.0044) and BED (10) of 90 Gy at the isocenter (HR 3.465, 95%CI [1.656–6.706], *p* = 0.0017), respectively (Table 4).

### Fiducial marker insertion

Among the 286 procedures of the fiducial marker insertions, data were available for 280 procedures. The number of fiducial markers implanted in each patient was from 1 to 7 (median: 4). The number of fiducial markers at the start date of the SBRT was from 1 to 6 (median: 4). At the start date of the SBRT there were 918 fiducial markers out of a total of 1100 inserted. During the treatment, replanning was required in 3 patients due to inter-fractional migration of fiducial markers. Pneumothorax related to the insertion of the fiducial markers was observed in 3 patients (Grade 1: one patient, Grade 2: two patients). One patient showed Grade 2 tachycardia. No patients experienced Grade 3 or higher complications.

### Radiation pneumonitis

The median of the PTV was 52.4 cc (5.7–313 cc) in the 252 plans which were available for analysis. Lung volume was defined as the bilateral lung volume minus the PTV volume. The median of the lung volumes was 3093 cc (1325–6886 cc) in the 257 plans. The median of the mean lung dose (MLD) was 355 cGy (101–996 cGy) in 239 plans. The median of the percentage of lung volume receiving a dose of 5 Gy or more (V5) and a dose of 20 Gy or more (V20) were 19.7% (6.2–45.4%) in 228 plans and 5.0% (1.0–16.0%) in 246 plans, respectively.

Radiation related pneumonitis with the SBRT could be assessed for 275 treatments. Nine patients had Grade 3 radiation pneumonitis. Grade 2 or higher radiation pneumonitis were observed in 38 patients (Grade 2: 29, Grade 3: 9). No patients experienced Grade 4 or 5 radiation pneumonitis. One of nine patients with Grade 3 radiation pneumonitis developed Grade 5 infectious pneumonia. This male patient received 40 Gy to the PTVD_95_ in 4 fractions for a 2.5 cm diameter tumor at the left upper lobe. Here MLD, V5 and V20 were 494 cGy, 23.7% and 10.1%, respectively. This patient experienced Grade 3 radiation pneumonitis 2 months after the SBRT. Although the radiation pneumonitis was improved by steroid therapy, the patient subsequently developed cytomegalovirus pneumonia 4 months after the SBRT and passed away, likely due to immunosuppression caused by the steroids.

The results of the univariate and multivariate analyses of the rates of radiation pneumonitis are shown in Table 5. Among the 271 treatments in which it was possible to evaluate both radiation pneumonitis and margins from GTV to PTV, Grade 2 or higher radiation pneumonitis was observed in 32 (21.2%) of 151 treatments with margins from GTV to PTV ≥ 10 mm, and there were 5 (4.2%) of 120 treatments with margins < 10 mm. In the univariate analysis, a statistically significant difference was observed in the rates of Grade 2 or higher radiation pneumonitis between these two subgroups. There was also a statistically significant difference observed between the subgroups with BED (10) at the isocenter ≥ 90 Gy and BED (10) < 90 Gy (14.8% vs. 0.0%, *p* = 0.0154), but no significant difference between BED (10) ≥ 100 Gy at the isocenter and BED (10) < 100 Gy (14.7% vs. 11.5%, *p* = 0.4839, not listed in Table 5). In the multivariate analysis, significant risk factors for Grade 2 or higher radiation pneumonitis were GTV to PTV margin ≥ 10 mm (OR 6.479, 95% CI [2.558–19.988], *p* < 0.0001), BED (10) at the isocenter ≥ 90 Gy (OR not estimable (NE), 95%CI [1.803–NE], *p* = 0.0118) and lower lobe tumors (OR 2.281, 95%CI [1.042–5.063], *p* = 0.0392), respectively (Table 5).

**Table 5:** Univariate and multivariate analysis results of Grade 2 or higher radiation pneumonitis rate

Variables		Grade 2 ≥ Radiation Pneumonitis		Univariate Analysis		Multivariate Analysis	
		n	Rates (%)	OR (95%CI)	*p*-value	OR (95%CI)	*p*-value
T stage	T1a + T1b	189	13.2	Reference	-	Reference	-
	T2a + T2b	86	15.1	1.168 (0.552–2.377)	0.6760	1.381 (0.590–3.121)	0.4485
Histology	Adenocarcinoma	177	15.3	1.553 (0.698–3.817)	0.2902	1.492 (0.608–3.963)	0.3896
	SCC	77	10.4	Reference	-	Reference	-
Gender	Female	69	15.9	1.257 (0.567–2.632)	0.5600	1.298 (0.525–3.099)	0.5643
	Male	206	13.1	Reference	-	Reference	-
Location	Upper and Middle	160	11.3	Reference	-	Reference	-
	Lower	115	17.4	1.661 (0.834–3.329)	0.1481	2.281 (1.042–5.063)	0.0392
BED (10) IC (Gy)	≥90	256	14.5	NE (1.593–NE)	0.0154	NE (1.803–NE)	0.0118
	<90	19	0.0	Reference	-	Reference	-
Margins from GTV to PTV (mm)	≥10	151	21.2	6.185 (2.530–18.579)	<0.0001	6.479 (2.558–19.988)	<0.0001
	<10	120	4.2	Reference	-	Reference	-

*BED* biologically effective dose, *CI* confidence interval, *GTV* gross tumor volume, *HR* hazard ratio, *IC* isocenter, *NE* not estimable, *PTV* planning target volume, *SCC* squamous cell carcinoma

### Other treatment related toxicities

One patient experienced Grade 3 dermatitis, and 10 patients reported Grade 2 thoracic wall pain. No other toxicities were recorded.

## Discussion

Guckenberger et al. have pointed out that image-guidance, gating, and real-time tracking can improve accuracy in pulmonary stereotactic body radiotherapy [25]. They investigated the required safety margins in SBRT by pre- and post-treatment cone-beam CT imaging in 43 patients, and found that stereotactic patient positioning and image-guidance based on the bony anatomy required safety margins of 12 mm and 9 mm, respectively. Four-dimensional image-guidance targeting of the tumor itself and intra-fractional tumor tracking made it possible to reduce margins to < 5 mm and < 3 mm, respectively. That study suggested that additional safety margins are required to compensate for breathing motion. Shimizu et al. and others have shown that the RTRT system can reduce the additional PTV margins for interfractional as well as intrafractional target motion taking account of baseline shift/drift and fluctuations in the amplitude during the treatment [12, 13, 26].

Inoue et al. have reported the experience with RTRT in a single institution where the 5-year LC rate was 78% and the 5-year OS rate was 64% for 109 patients (79 T1N0M0 and 30 T2N0M0) with a median follow-up period of 25 months (range, 4 to 72 months) [20]. In the present multi-institutional retrospective study, the OS rates for Stage I and IIA NSCLC were 75% and 64% at 2 and 3 years respectively. This is consistent with the 70% (95% CI: 67–72%) OS rates at 2 years for 3201 patients in a systematic review [27] and a 47% 3-years OS of 582 patients in the recent patterns-of-care and patterns-of-outcome analysis of SBRT for Stage I NSCLC [28].

A recent review article summarized the LC rates of SBRT for Stage I NSCLC and showed that the 3-year local control rates were widely distributed, from 40% to 92%, in studies which had a longer than 2-year follow-up [29]. The wide variation in LC rates may be ascribed to the difficulty of ensuring a uniform definition of LC because of the radiological changes after SBRT for periods of years [30]. The LC rates for Stage I and IIA NSCLC was 75% at 3 years in this series. This is consistent with the recent patterns-of-care and patterns-of-outcome analysis which showed 3-years of freedom from local progression of 79.6% [28]. A significant difference in the LC rate was found at a BED (10) of 90 Gy at the isocenter but not at 100 Gy or higher. The threshold for a high LC in the previous studies of SBRT for stage I NSCLC has been reported to be 100 Gy or higher in BED (10) at the isocenter [4].

A recently published report, in which local tumor control probability (TCP) in SBRT was evaluated, showed that a strong dose–response relationship was observed in the primary NSCLC and the dose to achieve 90% TCP was BED (10) at the isocenter > 176 Gy [31]. According to the dose response curve demonstrated in that report, BED (10) of 90 Gy at the isocenter would result in a local control of about 75%, matching the results of this study. As both BED (10) of 90 Gy and 100 Gy were in the steep part of the dose response curve, the difference between the threshold for LC BED (10) of 90 Gy in this study and 100 Gy in Onishi et al.’s report [4] could arise from differences in heterogeneity (tumor, patients, treatment and other factors) between these studies.

In our multivariate analysis, female was a significant predictive factor for OS and adenocarcinoma was significant for the LC rate. It is still not commonly agreed whether tumor histology is related to clinical outcomes in NSCLC treated with SBRT. Some studies have reported an absence of statistically significant differences in the survival or recurrence rates of adenocarcinomas and SCC [32, 33]. Matsuo et al. analyzed 101 patients with histologically confirmed stage I NSCLC who underwent SBRT [34], and reported that females had a significantly better prognosis than males and that histology was less significant. They suggested that this result may be caused by the proportion of lung adenocarcinomas in females being higher than in males. In our study, the situation was similar, with female patients having a significantly higher proportion of adenocarcinomas (Additional file 1) and a higher OS rate than males. In the LC rate, gender differences were not statistically significantly different but adenocarcinoma was a statistically significant predictive factor. One possible explanation for this is that gender differences in tumor histology may result in higher survival rates in females and higher LC rates in adenocarcinomas. Future study will be needed to further investigate the relationships between gender and tumor histology.

The potential benefit of the RTRT system strongly depends on the reproducibility of the position of the marker and the target volume. The relationship between the marker and the tumor position has been investigated in detail. As there is a learning curve for the insertion of fiducial markers through bronchial fiberscopy [14, 35] a strictly observed verification routine before treatment is mandatory [36], clinical training of the pulmonologists and radiation oncologists must be conducted in all institutions which install the RTRT system. The present study showed that there were no differences in the OS and LC rates among the different institutions. This absence of differences does not preclude a dependence of the clinical outcome on the insertion techniques but is encouraging and implies that any effect of a learning curve is minimal provided that proper training of the staff is available.

The distance between the fiducial marker and the target volume may change more in the lower lobe than in the middle or upper lobes during irradiation [37]. However, we did not see any difference in the OS and LC rates among the upper, middle, and lower lobes. Again here, any similarity in the OS and LC rates does not preclude a dependence of the clinical outcome to arise from differences in the location of the tumor but the effect as determined in this study is suggested to be minimal.

It is well known that the risk of radiation pneumonitis is correlated to the mean lung dose or other parameters which are related to dose volume statistics [38–43]. The RTRT is expected to reduce the volume of normal lung tissue which receives radiation doses that could give rise to the development of radiation pneumonitis. In the present study, Grade 2, 3, 4, and 5 radiation pneumonitis was experienced by 29 (10.2%), 9 (3.2%), 0, and 0 patients, respectively among 283 patients. Inoue et al. have reported the RTRT experience in a single institution and found that Grade 2, 3, 4, and 5 radiation pneumonitis was experienced by 15 (13.8%), 3 (2.8%), 0, and 0 patients, respectively in 109 patients [20]. In a Japanese multi-institutional prospective trial of SBRT 48 Gy was prescribed at the isocenter in 4 fractions for T1N0M0 NSCLC [5], the Grade 3, 4, and 5 radiation pneumonitis incidence was as follows: 9 (5.9%), 1 (0.6%), and 0 patients, respectively in 169 patients (Grade 2 incidence was data not shown). Although attention must be paid to compare the results from a retrospective study with those from a prospective study, the low complication rate in this study is consistent with other SBRT studies. We have however seen one (0.3%) Grade 5 adverse event, which is consistent with the Nagata et al. report in which there were 14 (0.6%) Grade 5 complications among 1111 patients who were treated with lung SBRT [44]. Since the complication rate has been reported to be very low in other SBRT studies, it is not certain whether RTRT was effective to reduce the complication rate below that of other SBRT technologies. Subgroup analyses demonstrated that there were no significant differences in the OS and the LC rates between margins from GTV to PTV ≥ 10 mm and margins < 10 mm, whereas the subgroup with margins ≥ 10 mm showed higher rate of Grade 2 or higher radiation pneumonitis. A prospective study will be required to determine whether RTRT with margins from GTV to PTV < 10mm would allow increasing the dose to the tumor and reduce the risk of radiation pneumonitis.

## Conclusions

This multi-institutional retrospective study of SBRT using a RTRT system for stage I and IIA NSCLC showed that the OS and LC rates were consistent with the recent patterns-of-care and patterns-of-outcome analysis of SBRT. The subgroup analyses revealed that smaller margins from GTV to PTV did not worsen the OS and the LC rates, while reducing the risk of radiation pneumonitis. A prospective study will be required to evaluate SBRT using an RTRT system with margins from GTV to PTV < 10mm.

## Additional file

Additional file 1: Table S1. Number of tumors by histology in male and female. (DOCX 32 kb)

## Abbreviations

BED: Biologically effective dose
CI: Confidence interval
CTV: Clinical target volume
Fr: Fractions
GTV: Gross tumor volume
Gy: Gray
HR: Hazard ratio
LC: Local control
MLD: Mean lung dose
MV: Megavoltage
NE: Not estimable
NSCLC: Non-small cell lung cancer
OR: Odds ratio
OS: Overall survival
PTV: Planning target volume
RTRT: Real-time tumor-tracking radiotherapy
SBRT: Stereotactic body radiotherapy
SCC: Squamous cell carcinoma
