# Supplementary material for: Clinical outcomes of stage I and IIA non-small cell lung cancer patients treated with stereotactic body radiotherapy using a real-time tumor-tracking radiotherapy system
Source: Radiat Oncol. 2017 Jan 5;12:3. doi: 10.1186/s13014-016-0742-3 (PMC5217432; doi:10.1186/s13014-016-0742-3)
Supplement: Additional file 1: Table S1. — Number of tumors by histology in male and female. (DOCX 32 kb) [file 13014_2016_742_MOESM1_ESM.docx]

**Additional Table 1.** Number of tumors by histology in male and female.

|  | | Histology | | *p*-value* |
| --- | --- | --- | --- | --- |
|  |  | Adenocarcinoma | SCC |  |
| Gender | Male (n=198) | 124 | 74 | <0.0001 |
|  | Female (n=67) | 61 | 6 |  |

SCC: squamous cell carcinoma. *chi-square test.
